# A model-based spectral directional approach reveals the long-term impact of COVID-19 on cardiorespiratory control and baroreflex

**DOI:** 10.1186/s12938-024-01327-8

**Published:** 2025-02-03

**Authors:** Beatrice Cairo, Francesca Gelpi, Vlasta Bari, Martina Anguissola, Pavandeep Singh, Beatrice De Maria, Marco Ranucci, Alberto Porta

**Affiliations:** 1 Department of Biomedical Sciences for Health, University of Milan, Milan, Italy; 2 Department of Cardiothoracic, Vascular Anesthesia and Intensive Care, IRCCS Policlinico San Donato, San Donato Milanese, Italy; 3 IRCCS Istituti Clinici Scientifici Maugeri, Milan, Italy

**Keywords:** SARS-CoV-2, Heart rate variability, Cardiorespiratory coupling, Causality, Closed-loop cardiovascular interactions, Autonomic nervous system, Active standing

## Abstract

**Background:**

Coronavirus disease 19 (COVID-19) patients might develop sequelae after apparent resolution of the infection. Autonomic dysfunction and baroreflex failure have been frequently reported. However, the long-term effect of COVID-19 on cardiorespiratory and cardiovascular neural controls has not been investigated with directional approaches able to open the closed-loop relationship between physiological variables.

**Methods:**

A model-based causal spectral approach, namely causal squared coherence (CK^2^), was applied to the beat-to-beat variability series of heart period (HP) and systolic arterial pressure (SAP), and to the respiratory signal (RESP) acquired at rest in supine position and during active standing (STAND) in COVID-19 survivors 9 months after their hospital discharge. Patients were categorized according to their need of ventilatory support during hospitalization as individuals that had no need of continuous positive airway pressure (noCPAP, *n* = 27), need of continuous positive airway pressure in sub-intensive care unit (CPAP, *n* = 14) and need of invasive mechanical ventilation in intensive care unit (IMV, *n* = 8).

**Results:**

The expected decrease of the strength of the HP-RESP dynamic interactions as well as the expected increase of the dependence of HP on SAP along baroreflex during STAND was not observed and this result held regardless of the severity of the disease, namely in noCPAP, CPAP and IMV cohorts. Regardless of the experimental condition, spectral causality markers did not vary across groups either.

**Conclusions:**

CK^2^ markers, in association with an orthostatic challenge, were able to characterize the impairment of cardiorespiratory control and baroreflex in COVID-19 patients long after acute infection resolution and could be exploited to monitor the evolution of the COVID-19 patients after hospital discharge.

## Background

In late 2019, a novel coronavirus named severe acute respiratory syndrome coronavirus 2 (SARS-CoV-2) emerged in Wuhan, China, and has since spread throughout the world, causing the coronavirus disease 2019 (COVID-19). COVID-19 features a great variety of symptoms including those of respiratory origin [1, 2]. Especially in older patients with comorbidities, the pathology can evolve into severe disease associated with respiratory distress syndrome and/or cardiac injury, in a context of multi-organ dysfunction and hyperinflammatory immune response [3–6]. Therefore, in a percentage of all COVID-19 patients, hospitalization and even admission in intensive care unit (ICU) are required because COVID-19-induced respiratory failure might necessitate invasive mechanical ventilation (IMV) [7].

Since the beginning of the COVID-19 pandemic, it has become evident that many people recovering from a SARS-CoV-2 infection might develop sequelae after apparent resolution of the initial infection, with no alternative cause [2, 8]. It is known [9] that COVID-19 affects the autonomic nervous system (ANS) by damaging either directly [10, 11] or indirectly [12] its central structures. Unexpected autonomic responses suggest the ANS involvement in COVID-19 patients even during their ICU stay [13]. The ANS impairment is likely to be responsible for the development of symptoms of dysautonomia [14–17] and baroreflex failure [18, 19]. The assessment of the impairment of the autonomic function and baroreflex control is mainly based on the evaluation of univariate markers derived from spontaneous changes of heart period (HP) and systolic arterial pressure (SAP) [19–22] and on the computation of bivariate markers of baroreflex sensitivity derived from HP and SAP variability series [18, 19]. In addition to these traditional approaches, causality analysis [23] has been utilized to assess vagal cardiac control and baroreflex regulation, respectively, from the analysis of the dynamic interactions between HP variability and respiration (RESP) [24–30] and between HP and SAP changes [24–34], especially in association with a postural challenge inducing a modification of the venous return and subsequent autonomic response [25–33]. The main advantage of causality analysis compared to more traditional univariate and transfer function-based techniques is to account for directionality, thus allowing the separation of the feedforward pathway from the feedback one in the closed-loop interactions [34–39], and to assure an intrinsic normalization preventing the dependence of markers on the amplitude of fluctuations. Moreover, since HP-RESP and HP-SAP causality analyses have been extensively tested in healthy subjects during orthostatic challenges [25–28, 31–33], the impact of active standing (STAND) on causality markers is well-known, thus favoring the detection of a possible impairment in individuals featuring a non-physiological response to STAND. An additional advantage of causality analysis, when it is carried out via spectral methods, is the possibility to evaluate indexes in specific frequency bands typical of the vagal cardiac control, namely in the high frequency (HF) band (from 0.15 to 0.4 Hz) [40, 41], and of the baroreflex control, namely in the low frequency (LF) band (from 0.04 to 0.15 Hz) [37, 38]. The application of causality markers to assess the long-term impact of COVID-19 might provide additional insight compared to more usual univariate approaches based on the exclusive analysis of HP variability [42].

Thus, in the present study we apply a model-based spectral causality approach, namely causal squared coherence (CK^2^) [43, 44] to study the dynamical interactions between RESP and HP variability and between SAP and HP variabilities in COVID-19 survivors assessed after 9 months from the hospital discharge and classified according to the severity of their condition during hospitalization. Traditional squared coherence (K^2^) was computed as well. We hypothesize that COVID-19 might produce long-term modifications of cardiorespiratory coupling and baroreflex and results might depend on the severity of the COVID-19. Causality analysis was carried out along the feedforward and feedback pathways of the HP-SAP and HP-RESP closed-loop relations to facilitate the association of the findings with specific physiological mechanisms. STAND was utilized as a challenge to stimulate the response of the ANS.

## Results

In Table 1, the longer length of stay in hospital and ICU stressed the more severe condition of CPAP group compared to the noCPAP cohort and of IMV group compared to both noCPAP and CPAP cohorts, respectively. No additional significant difference was found with the notable exception of a less frequent use of betablockers in the CPAP cohorts compared to the noCPAP and IMV ones.

**Table 1:** Demographic and clinical characteristics of COVID-19 patients

Variable	noCPAP (*n* = 27)	CPAP (*n* = 14)	IMV (*n* = 8)
Age [yrs]	63.3 ± 9.1	63.4 ± 11.5	61.8 ± 10.3
Gender [male]	18 (67)	10 (71)	6 (75)
BMI [kg·m^−2^]	28.4 ± 3.7	30.3 ± 9.1	30.2 ± 3.5
Length of hospital stay [days]	16.1 ± 12.5	25.6 ± 17.3#	24.4 ± 10.9#
Length of ICU stay [days]	0 ± 0	0 ± 0	10.4 ± 8.2#,§
Congestive heart failure	0 (0)	0 (0)	0 (0)
Recent myocardial infarction	0 (0)	1 (7.1)	0 (0)
Previous cerebrovascular events	0 (0)	1 (7.1)	1 (12.5)
Diabetes	4 (14.8)	6 (42.9)	2 (25.0)
COPD	1 (3.7)	0 (0)	0 (0)
Hypertension	20 (74.1)	9 (64.3)	4 (50.0)
ACE inhibitors	11 (40.7)	6 (42.9)	3 (37.5)
Beta-blockers	13 (48.1)	1 (7.1)#	3 (37.5)§
Diuretics	6 (22.2)	5 (35.7)	3 (37.5)
Calcium antagonist	4 (14.8)	6 (42.9)	1 (12.5)

Continuous variables are presented as mean ± standard deviation. Categorical variables are presented as number (percentage). The symbol # and § indicates a significant difference with *p* < 0.05 compared to the noCPAP and CPAP groups respectively

noCPAP = group who did not undergo continuous positive airway pressure; CPAP = group who underwent continuous positive airway pressure; IMV = group who underwent invasive mechanical ventilation; BMI = body mass index; ICU = intensive care unit; COPD = chronic obstructive pulmonary disease; ACE = angiotensin-converting enzyme.

Univariate time and frequency domain HP, SAP, and RESP markers are listed in Table 2. STAND induced tachycardia in all the groups (*i.e.*, noCPAP, CPAP, IMV), while σ^2^_HP_ did not vary. HF_HP_ decreased and LF_SAP_ increased during STAND exclusively in the noCPAP group. μ_SAP_, σ^2^_SAP_, and f_RESP_ were stable with STAND and this finding held regardless of the group. Between-group differences were not observed with the notable exception of HF_HP_ being smaller in CPAP compared to noCPAP at REST.

**Table 2:** Univariate time and frequency domain indexes derived from HP, SAP, and RESP series in COVID-19 patients

**Variable**	**noCPAP** (n = 27)		**CPAP** (n = 14)		**IMV** (n = 8)	
	**REST**	**STAND**	**REST**	**STAND**	**REST**	**STAND**
μ_HP_ [ms]	884 ± 130	798 ± 130*	826 ± 122	777 ± 105*	831 ± 94	758 ± 100*
σ^2^_HP_ [ms^2^]	749 ± 487	683 ± 468	559 ± 432	393 ± 470	840 ± 666	546 ± 484
HF_HP_ [ms^2^]	162 ± 175	64 ± 59*	55 ± 45#	56 ± 77	111 ± 113	52 ± 47
μ_SAP_ [mmHg]	138 ± 14	143 ± 23	138 ± 22	128 ± 17	140 ± 27	145 ± 23
σ^2^_SAP_ [mmHg^2^]	28.5 ± 20.7	40.6 ± 29.1	30.9 ± 26.2	32.0 ± 19.2	36.7 ± 26.8	27.7 ± 23.5
LF_SAP_ [mmHg^2^]	4.3 ± 2.6	9.9 ± 10.3*	4.4 ± 4.8	9.0 ± 10.2	5.2 ± 4.2	6.0 ± 6.4
*f*_RESP_ [bpm]	16.1 ± 2.6	16.4 ± 1.9	15.8 ± 2.8	15.1 ± 2.1	15.3 ± 2.0	15.3 ± 1.5

noCPAP = group who did not undergo continuous positive airway pressure; CPAP = group who underwent continuous positive airway pressure; IMV = group who underwent invasive mechanical ventilation; REST = at rest in supine position; STAND = during active standing; HP = heart period; SAP = systolic arterial pressure; μ_HP_ = HP mean; σ^2^_HP_ = HP variance; μ_SAP_ = SAP mean; σ^2^_SAP_ = SAP variance; HF = high frequency; HF_HP_ = HP power in the HF band expressed in absolute units; LF = low frequency; LF_SAP_ = SAP power in the LF band expressed in absolute units; f_RESP_ = respiratory frequency. Variables are presented as mean ± standard deviation. The symbol * indicates a significant difference with *p* < 0.05 compared to REST within the same group. The symbol # indicates a significant difference with *p* < 0.05 compared to noCPAP within the same experimental condition.

**Fig. 1:**
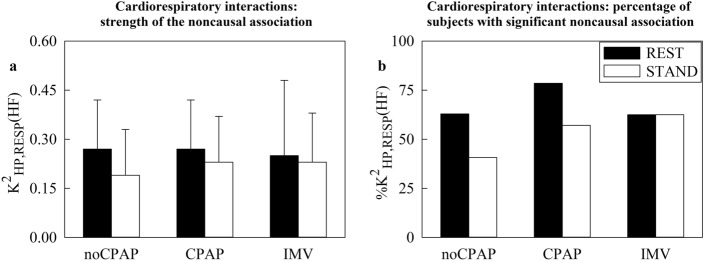
The vertical grouped error bar graph reports ${\text{K}}_{{\text{RESP}},{\text{HP}}}^{2}(\text{HF})$ in (**a**) and the vertical grouped bar graph shows ${\left[\kern-0.15em\left[ {\% {\text{K}}} \right]\kern-0.15em\right]}_{({\text{RESP}},{\text{HP}})}^{2} ({\text{HF}})$ in (**b**) as a function of the cohort (i.e., noCPAP, CPAP and IMV) at REST (black bars) and during STAND (white bars).

In Fig. 1, the vertical grouped error bar graph shows ${\text{K}}_{\text{RESP},\text{HP}}^{2}(\text{HF})$ (Fig. 1a) and the vertical group bar graph reports ${\text{\%K}}_{\text{RESP},\text{HP}}^{2}(\text{HF})$ (Fig. 1b). Markers are reported as a function of the groups (*i.e.*, noCPAP, CPAP and IMV) and experimental condition (REST: back bars; STAND: white bars). Neither ${\text{K}}_{\text{RESP},\text{HP}}^{2}(\text{HF})$ nor ${\text{\%K}}_{\text{RESP},\text{HP}}^{2}(\text{HF})$ varied with group and experimental condition.

**Fig. 2:**
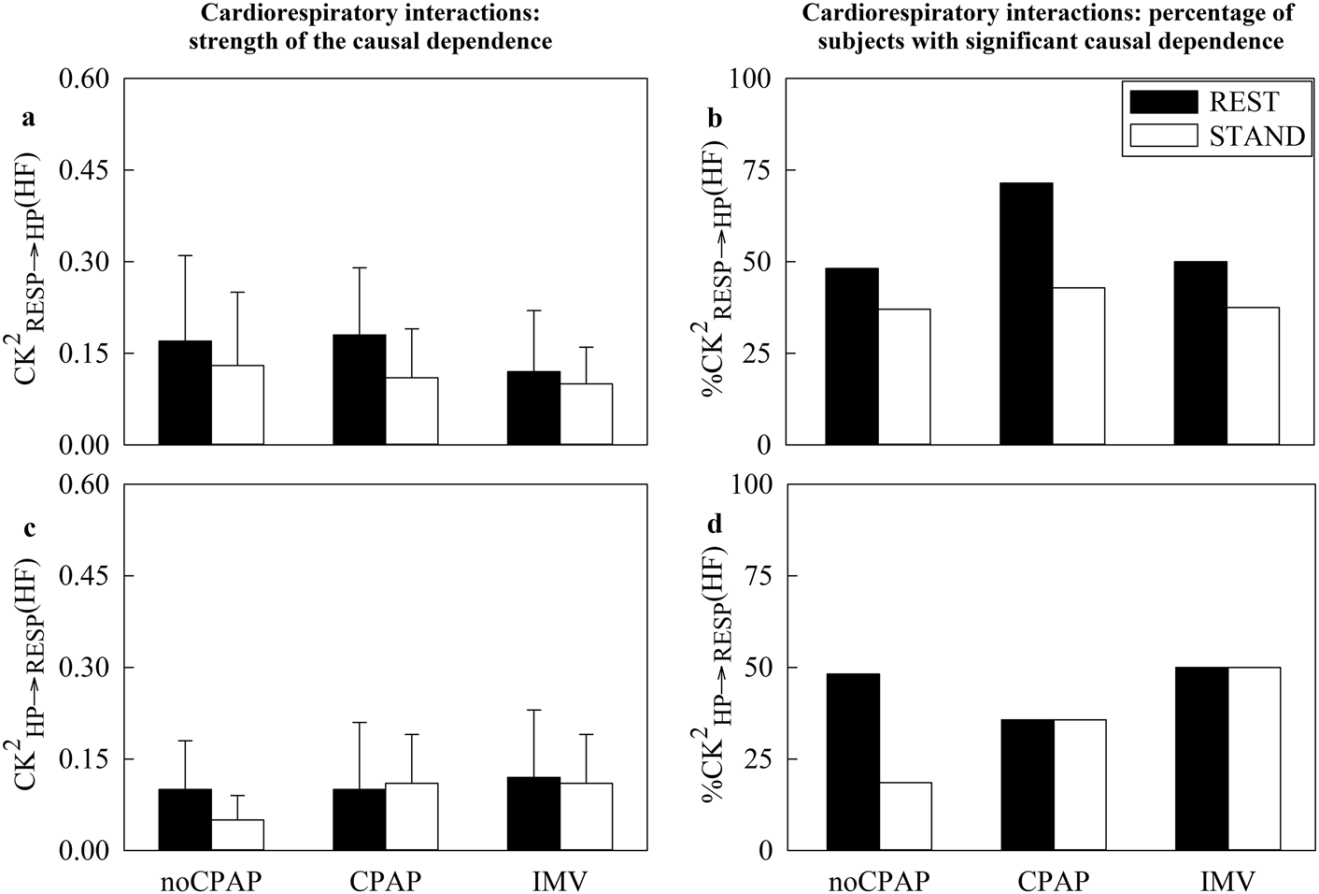
The vertical grouped error bar graphs report ${\text{CK}}_{{\text{RESP}}\to {\text{HP}}}^{2}({\text{HF}})$ in (**a**) and ${\text{CK}}_{{\text{HP}}\to {\text{RESP}}}^{2}({\text{HF}})$ in (**c**), while the vertical grouped bar graphs show ${\text{\%CK}}_{{\text{RESP}}\to {\text{HP}}}^{2}({\text{HF}})$ in (**b**) and ${\left[\kern-0.15em\left[ {\% {\text{CK}}} \right]\kern-0.15em\right]}_{({\text{HP}}{\to}{\text{RESP}})}^2 ({\text{HF}})$ in (**d**) as a function of the cohort (i.e., noCPAP, CPAP and IMV) at REST (black bars) and during STAND (white bars).

In Fig. 2, the vertical grouped error bar graphs show ${\text{CK}}_{\text{RESP}\to \text{HP}}^{2}(\text{HF})$ (Fig. 2a) and ${\text{CK}}_{\text{HP}\to \text{RESP}}^{2}(\text{HF})$ (Fig. 2c), while the vertical grouped bar graphs depict ${\text{\%CK}}_{\text{RESP}\to \text{HP}}^{2}(\text{HF})$ (Fig. 2b) and ${\text{\%CK}}_{\text{HP}\to \text{RESP}}^{2}(\text{HF})$ Fig. 2d). Markers are reported as a function of the groups and experimental conditions as in Fig. 1. Regardless of time direction of the interactions and type of the markers, no between-group and between-experimental condition differences were observed.

**Fig. 3:**
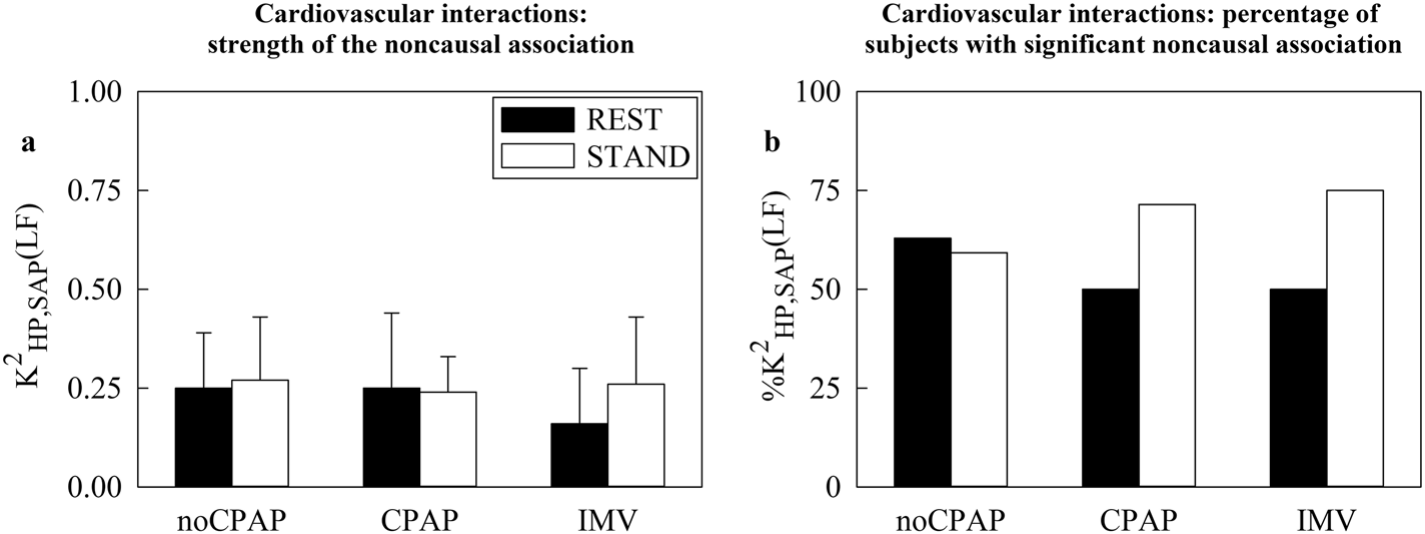
The vertical grouped error bar graph reports ${\text{K}}_{{\text{SAP}},{\text{HP}}}^{2}({\text{LF}})$ in (**a**) and the vertical grouped bar graph shows ${\left[\kern-0.15em\left[ {\% {\text{K}}} \right]\kern-0.15em\right]}_{({\text{SAP}},{\text{HP}})}^2 ({\text{LF}})$ in (**b**) as a function of the cohort (i.e., noCPAP, CPAP and IMV) at REST (black bars) and during STAND (white bars).

Figure 3 has the same structure as Fig. 1, but it shows ${\text{K}}_{\text{SAP},\text{HP}}^{2}(\text{LF})$ (Fig. 3a) and ${\text{\%K}}_{\text{SAP},\text{HP}}^{2}(\text{LF})$ (Fig. 3b). Neither ${\text{K}}_{\text{SAP},\text{HP}}^{2}(\text{LF})$ nor ${\text{\%K}}_{\text{SAP},\text{HP}}^{2}(\text{LF})$ varied with group and experimental condition.

**Fig. 4:**
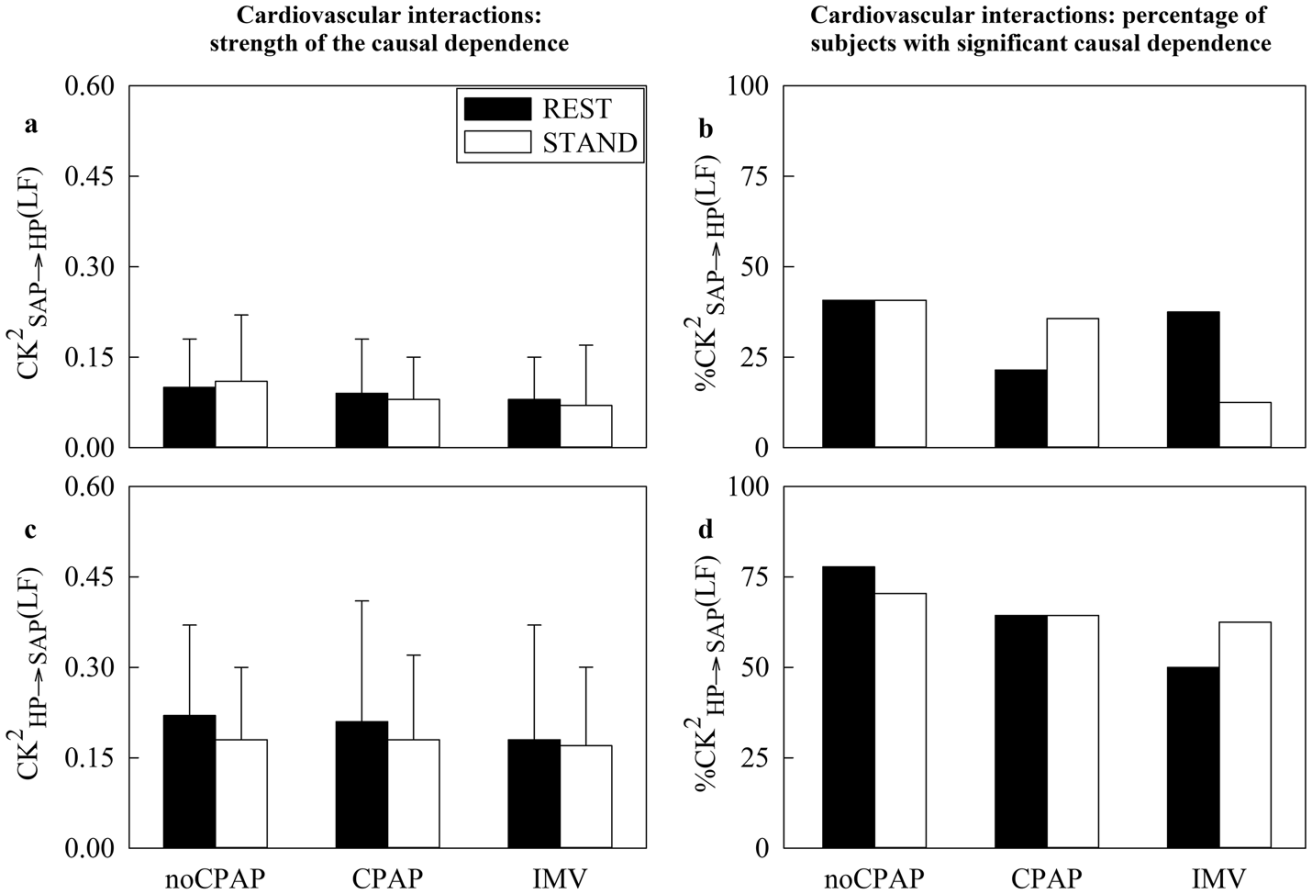
The vertical grouped error bar graphs report ${\text{CK}}_{{\text{SAP}}\to {\text{HP}}}^{2}({\text{LF}})$ in (**a**) and ${\text{CK}}_{{\text{HP}}\to {\text{SAP}}}^{2}({\text{LF}})$ in (**c**), while the vertical grouped bar graphs show ${\text{\%CK}}_{{\text{SAP}}\to {\text{HP}}}^{2}({\text{LF}})$ in (**b**) and ${\left[\kern-0.15em\left[ {\% {\text{CK}}} \right]\kern-0.15em\right]}_{({\text{HP}}{\to}{\text{SAP}})}^2 ({\text{LF}})$ in (**d**) as a function of the cohort (i.e., noCPAP, CPAP and IMV) at REST (black bars) and during STAND (white bars).

Figure 4 has the same structure as Fig. 2, but it shows ${\text{CK}}_{\text{SAP}\to \text{HP}}^{2}(\text{LF})$ (Fig. 4a), $\%C{\text{K}}_{\text{SAP}\to \text{HP}}^{2}(\text{LF})$ (Fig. 4b), ${\text{CK}}_{\text{HP}\to \text{SAP}}^{2}(\text{LF})$ (Fig. 4c), and ${\text{\%CK}}_{\text{HP}\to \text{SAP}}^{2}(\text{LF})$ (Fig. 4d). Regardless of time direction of the interactions and type of the markers, no between-group and between-experimental condition differences were observed.

In summary, none of the cardiorespiratory, or cardiovascular, markers of association, as expressed in absolute value or percentage of subjects with significant degree of association, varied across groups and experimental conditions (Figs. 1, 3). This finding held even for cardiorespiratory and cardiovascular indexes assessing casual dependences (Figs. 2, 4).

## Discussion

The main results of the study can be summarized as follows: i) a model-based spectral causality approach, such as CK^2^, was found to be useful to detect the derangement of cardiorespiratory and cardiovascular controls in COVID-19 patients; ii) the impairment took the form of the lack of response of HP-RESP control and of baroreflex to STAND; iii) the unresponsiveness of the closed-loop HP-RESP control and baroreflex was evident even in the noCPAP cohort; iv) markers describing the closed-loop RESP-HP and SAP-HP regulations did not vary with the severity of COVID-19.

### A spectral causality approach is useful to detect the derangement of cardiorespiratory and cardiovascular controls in COVID-19 patients long after their acute infection period

Directional analysis based on the evaluation of statistical dependences is commonly applied to spontaneous fluctuations of HP and SAP and recordings of RESP activity to characterize cardiovascular and cardiorespiratory control mechanisms [24–34]. This analysis is superior to the more traditional tools based on cross-spectral density function [39, 40] because the metrics exploited are asymmetric under the reversal of the role of the two series, thus allowing one to set a causal direction from a driver to a presumed responder [23]. Among directional tools, the ones in frequency domains assure the remarkable advantage of explicitly accounting for the time scales of the mechanism under scrutiny, thus being particularly specific. For example, when assessing cardiorespiratory interactions the respiratory band (*i.e.*, HF) is particularly interesting [35, 39, 40] as well as the LF band when assessing the cardiac arm of the baroreflex [37–39]. An additional advantage of this analysis is the intrinsic normalization typical of markers computing the degree of association between variables that limits the dependence of these indexes on the absolute value of the changes of the physiological variables. This study confirms the value of a model-based spectral causality tool, such as CK^2^ [43, 44], in a clinical context such as the characterization of COVID-19 patients long after their hospital discharge (*i.e.*, 9 months). Indeed, CK^2^, in association with the application of an orthostatic challenge such as STAND, suggested that COVID-19 has a profound impact on cardiorespiratory and cardiovascular control mechanisms, evident even in the noCPAP cohort. Although the missed response to STAND was detectable even using more traditional univariate indexes in the CPAP and IMV groups, and classical K^2^ markers in all the cohorts, the missing indication about the direction of interactions make these indexes less suitable for interpreting physiological modifications. For example, our analysis supports a profound impact of COVID-19 on neural autonomic circuits, *e.g.*, those governing the baroreflex pathway from SAP to HP, while the mechanical feedforward pathway from HP to SAP, more related to ventricular contractility and vascular properties of arterial tree, appears to be more preserved.

### COVID-19 impairs the response of closed-loop HP-RESP control to STAND

Significant closed-loop interactions are commonly detected between spontaneous fluctuations of HP and RESP using model-based [24, 51, 52] and model-free [36, 53] approaches. The link from RESP to HP is the result of the activity of respiratory centers modulating vagal activity to the heart [40, 54], even though contributions of sympathetic circuits limiting respiratory sinus arrhythmia are detected [55]. In the healthy population the sympathetic activation and vagal withdrawal associated with the decline of the venous return during STAND [56–59] reduced the dependence of HP on RESP [25–27, 60]. It has been suggested that the latency of the cardiac beat just preceding the inspiratory onset to the respiratory onset is fixed (about 0.5 s), thus indicating that the heart can contribute to initiating the respiratory activity [61, 62]. The significance of the link from HP to RESP, detected under spontaneous and control respiration in healthy subjects, confirmed the relevance of the pathway from HP to RESP [63, 64], even using causal approaches [24, 36, 51–53]. The vagal arm of the baroreflex governing HP modifications in response to changes of AP induced by the cardiac beat just preceding the respiratory onset could contribute to mediating this relationship [65, 66]. Again, since sympathetic activation and vagal withdrawal associated with STAND reduced baroreflex sensitivity in healthy subjects [56], the strength of the dependence of RESP on HP is expected to decrease during STAND [36]. Since we observed the lack of a significant impact of STAND on both ${\text{CK}}_{\text{RESP}\to \text{HP}}^{2}(\text{HF})$ and ${\text{CK}}_{\text{HP}\to \text{RESP}}^{2}(\text{HF})$, we conclude that COVID-19 impairs closed-loop HP-RESP relationship. The lack of response to STAND suggests the limited ability of sympathetic control in reducing the efficiency of the central respiratory pattern generator in modulating vagal outflow. The negligible impact of STAND was corroborated by the unvaried percentage of the rejections of the null hypothesis of HP-RESP uncoupling during STAND compared to REST in both causal directions. Remarkably this conclusion held regardless of the severity of COVID-19 and it was evident even in the noCPAP group.

### COVID-19 impairs the response of closed-loop HP-SAP control to STAND

It is well-known that HP and SAP interact in closed loop [34, 37–39]. The pathway from SAP to HP is the consequence of the cardiac arm of the baroreflex responsible for limiting the variability of SAP with suitable variations of HP [66–68]. The baroreflex is a vagally-mediated reflex [69] the sensitivity of which is reduced during sympathetic activation and vagal withdrawal observed during orthostatic challenges [56, 70–72], while its degree of engagement increases [25, 26, 31–33, 73, 74]. Consequently, in healthy population STAND is expected to increase the degree of dependence of HP on SAP. On the reverse causal direction SAP is driven by changes of HP through the so-called mechanical feedforward pathway [37–39]. This pathway is the result of the balance between two opposite SAP tendencies occurring in the response of the same HP prolongation, namely a positive contribution resulting from the Frank-Starling law and the more important left ventricular filling, and a negative one resulting from the longer diastolic decay diminishing AP [37]. In the healthy subjects, tachycardia and sympathetic activation, induced by orthostatic challenge, reduce ventricular filling and limit diastolic runoff respectively, thus producing opposite influences on the mechanical feedforward pathway and leaving the degree of association from HP to SAP unvaried [31–33, 74]. Since in the present study we observed that ${\text{CK}}_{\text{SAP}\to \text{HP}}^{2}(\text{LF})$ and ${\text{CK}}_{\text{HP}\to \text{SAP}}^{2}(\text{LF})$ did not change during STAND, we conclude that baroreflex is impaired, while the mechanical feedforward pathway seems to be preserved, at least in terms of the balance of the impact between mechanical properties of the left ventricle and vascular properties of the arterial tree. The missed engagement of the baroreflex during STAND might explain the frequent observation of orthostatic intolerance and episodes of exaggerated modifications of AP in response to stimuli in post-acute COVID-19 cohorts. The impaired response of baroreflex to STAND was supported by the unvaried percentage of the rejections of the null hypothesis of HP-SAP uncoupling during STAND compared to REST. Remarkably, the lack of engagement of the baroreflex during STAND held regardless of the severity of COVID-19 and it was evident even in the noCPAP group.

### The impact of the COVID-19 severity on closed-loop HP-RESP and HP-SAP relationships

Post-acute COVID-19 syndrome is frequently associated with symptoms of ANS dysfunction such as postural orthostatic tachycardia, orthostatic intolerance, and exaggerated AP modifications in response to stimuli [75, 76]. Previous studies suggested that severe forms of COVID-19 are more frequently associated with sinus tachycardia at the hospital discharge [77], thus suggesting that ANS impairment might be linked to the severity of COVID-19. However, it is unknown whether long-term autonomic symptoms are more frequent in patients who experienced a more severe pathology. Markers derived from univariate analysis of HP and SAP series suggested that the noCPAP group responded to STAND, while more severe forms of COVID-19 (*i.e.,* CPAP and IMV groups) did not. Indeed, we detected the expected decrease of HF_HP_ and increase of LF_SAP_ with the orthostatic challenge [41, 56–59] exclusively in the noCPAP group. This finding supports the conclusion that dysautonomia associated with COVID-19 is less dramatic in noCPAP patients. However, we observed that markers characterizing the HP-RESP and HP-SAP dynamic relationship did not vary across either group or experimental condition, thus suggesting that the impairment of the cardiorespiratory coupling and baroreflex function is a typical feature of COVID-19 regardless of its degree of severity in hospitalized patients. Remarkably, the missing differentiation of the noCPAP group with respect to the CPAP and IMV cohorts occurring after a relatively long period of time (*i.e.*, 9 months after the hospital discharge) indicates a missing recovery from the autonomic dysfunction of the group featuring the lowest degree of severity. Given that symptoms of dysautonomia, including the impairment of the cardiorespiratory control and baroreflex, are frequently observed in post-acute COVID-19 syndrome (between one-third and a half of highly symptomatic COVID-19 patients), the missing recovery in noCPAP group might suggest that future health-care burden might be even greater than expected [78, 79].

### Physiological and clinical perspectives

A typical autonomic response is expected during STAND because of the reduction of the venous return to the heart associated with the change of posture. Therefore, the absence of this typical response driven by the activation of the autonomic nervous system in post-acute COVID-19 patients without signs of long COVID-19 stresses that COVID-19 has long-term effects above and beyond those that are considered the signature of long COVID-19. The results of this study stress that long-term effects might take the form of a subtle non-physiological autonomic response to postural challenge, and they are present even in patients with less severe forms of infection. The non-invasiveness of our protocol and its simplicity based on an intervention that can be applied very easily (i.e., active postural challenge) facilitates its implementation to test the state of the autonomic control in post-COVID 19 patients. Remarkably, if it was limited to the evaluation of cardiorespiratory interactions, the protocol could be applied even outside the well-controlled conditions of the laboratory and be based on the sole acquisition of the ECG from which a RESP signal can be easily derived [80]. Suitable forms of long-term surveillance based on this approach could contribute to limiting the consequence of COVID-19 pandemic and future burden of the healthcare system. In addition, the application of this methodology might contribute to the development of appropriate countermeasures specifically designed for post-acute COVID-19 patients to recover a more physiological autonomic response to the postural challenge.

### Limitations of the study

The first limitation of the study is the lack of a control population matched for age, sex, and risk factors with the cohorts of this study. This lack does not allow us to prove that the impairment of the cardiorespiratory control and baroreflex is the genuine effect of the COVID-19 or the consequence of other pathologies or disease states. However, even though our cohorts were not composed exclusively by healthy people who were infected by SARS-CoV-2, the clinical characteristics of the groups do not support the conclusion that the missing responses of cardiorespiratory and baroreflex controls to STAND is the trivial consequence of previous pathologies. The second limitation is that the assumption of clinical and demographic indistinguishability of noCPAP, CPAP and IMV groups is not fully verified. Indeed, the fraction of individuals taking betablockers was smaller in the CPAP group compared to noCPAP and IMV cohorts. This feature might have contributed to the smaller HF_HP_ at REST in the CPAP group compared to noCPAP one [55]. However, this difference should not be considered a confounding factor for causality markers from RESP to HP and vice versa given that both ${\text{CK}}_{\text{RESP}\to \text{HP}}^{2}(\text{HF})$ and ${\text{CK}}_{\text{HP}\to \text{RESP}}^{2}(\text{HF})$ did not vary across groups. We confirm the limited impact of betablockers on final conclusions by performing a linear mixed model analysis for repeated measures using as fixed effects the group and experimental condition and as a random effect the use of betablocker therapy. We observed that the betablocker therapy did not affected significantly either noncausal or causal indexes in our protocol. The third limitation is the small size of the groups, especially for the CPAP and IMV cohorts. This result is the consequence of the enrollment of the patients during the second wave of the COVID-19 pandemic that in Italy featured a rate of high severity cases less important than that during the first one. This limitation could be faced solely by merging our database with those of other hospitals provided that the same experimental protocol and data collection modality were followed.

## Conclusions

The present study suggests the validity of model-based causal spectral indexes to characterize autonomic dysfunction and baroreflex failure in post-acute COVID-19 patients. Spectral causal markers appear to be superior to more traditional variability-based indexes because they provide the possibility to assess directional dependence and disentangle the feedback pathway from the feedforward one in closed-loop interactions, thus linking markers to specific physiological mechanisms and favor interpretation of the results. The missed expected response of cardiorespiratory and baroreflex controls to STAND could be considered an hallmark of the status of the post-acute COVID-19 patients. The same markers could be exploited in the follow-up to track the effect of pharmacological treatment or nonpharmacological countermeasures applied to reduce the impact of dysautonomia. The study highlights the impact of COVID-19 on the autonomic function, and more specifically on cardiorespiratory control and baroreflex, and suggests that dysautonomia is present 9 months after hospital discharge and even in COVID-19 patients featuring the lowest degree of severity during hospitalization, namely the noCPAP group. Since the participants of our study did not specifically report typical symptoms of dysautonomia, the results support the notion that the autonomic control derangement is more frequent than previously reported in post-acute COVID-19 patients and it is an issue that requires careful monitoring in the future and specific tools for its detection. The study confirms the severity of the consequence of the COVID-19 infection, thus allowing us to foresee an increased future burden of the healthcare system whether strategies to cope with post-acute COVID-19 patients were not designed.

## Methods

### Experimental protocol

We enrolled patients who had been admitted to IRCCS Policlinico San Donato following SARS-CoV-2 infection as confirmed by polymerase chain reaction. Patients were classified according to the severity of their condition during hospitalization into three groups: patients with severe complications who were admitted to the ICU and underwent IMV, patients with moderate complications who were admitted to the sub-ICU where they underwent continuous positive airway pressure (CPAP), patients with light complications admitted in general care unit where they did not need either IMV or CPAP for the entire duration of their hospitalization (noCPAP). Exclusion criteria included history of non-sinus cardiac rhythm, pregnancy at the time of the enrolment and age less than 18 years. Demographic and clinical characteristics of the cohorts are reported in Table 1. Pathological state and pharmacological treatment reported in Table 1 were preexistent to the admission in the hospital and confirmed at the discharge. Patients were recalled in the IRCCS Policlinico San Donato 9 months after the hospital discharge (from October 2021 to March 2022) for data acquisition. The study was conducted according to the principles of the Declaration of Helsinki for medical research involving humans and approved by Comitato Etico Ospedale San Raffaele, Milan, Italy (protocol number 148/INT/2021; approval date: 15/09/2021). Written informed consent was obtained from all subjects. We acquired the electrocardiogram (ECG) from lead II through a commercial bioamplifier (BioAmp FE132, ADInstruments, Australia), the non-invasive continuous arterial pressure (AP) via a volume-clamped photoplethysmographic device positioned at finger-level (CNAP Monitor 500, CNSystems, Austria) and the RESP signal as obtained from respiratory chest movements sensed via a thoracic piezoelectric belt (Respiratory Belt Transducer, ADInstruments, Australia). Signals were sampled at 400 Hz via a commercial analog-to-digital device (PowerLab, ADinstruments, Australia). The experimental session consisted in recording signals for 10 min at rest in supine position (REST), followed by a further 15 min during STAND. Patients were instructed to remain silent for the duration of the protocol. A stabilization period of 5 min was allowed before starting the acquisition session at REST and before starting the analysis during STAND.

### Extraction of beat‑to‑beat variability series

The R-wave peaks were detected via a threshold-based algorithm on the ECG first derivative. The *n*th HP was estimated as the time interval between two consecutive R-wave peaks. The *n*th SAP was defined as the maximum of AP within the *n*th HP. The RESP signal was sampled at the first R-wave peak defining the onset of the *n*th HP and its value was taken as the *n*th RESP. HP was expressed in ms, SAP in mmHg and RESP signal in mV. Sequences of 256 consecutive HP, SAP and RESP values were randomly selected for each experimental session (*i.e.*, REST and STAND). The HP beat-to-beat variability series were checked in correspondence of values outside a user-defined range. This check allowed the identification of missing R-wave detections or arrhythmic beats. In the case of a missed cardiac beat the R-wave detection was inserted, and the HP, SAP and RESP series were updated. In case of isolated, or consecutive, arrhythmic beats, the correspondent values of HP, SAP and RESP were linearly interpolated using the most adjacent values associated with sinus cardiac beats. Values of HP, SAP and RESP series requiring interpolation were less than 5% of the total series length.

### Time and frequency domain indexes

In the time domain, we computed the mean and variance of HP and SAP series, namely μ_HP_, σ^2^_HP_, μ_SAP_ and σ^2^_SAP_. The indexes were expressed in ms, ms^2^, mmHg, and mmHg^2^ respectively. In the frequency domain, the beat-to-beat HP, SAP, and RESP series were fitted via an autoregressive (AR) model [45]. Parameters were estimated by solving the least squares identification problem via the Levinson–Durbin recursion [45] and the order was optimized via the Akaike information criterion in the range between 8 and 14 [46]. The power spectral density, estimated from the transfer function of the AR model and variance of the prediction error [45], was factorized into spectral components each relevant to real poles or pairs of complex and conjugated poles. The power associated with a spectral component was computed using the residue theorem [47]. The components were classified according to whether their central frequency belonged to the LF, or HF, band. The total power of SAP in the LF band (LF_SAP_), expressed in absolute units (mmHg^2^), was taken as a marker of sympathetic modulation directed to the vessels [48]. The total power of HP in the HF band (HF_HP_), expressed in absolute units (ms^2^), was taken as a marker of vagal modulation directed to the heart [40, 41]. The RESP frequency (f_RESP_) was estimated as the frequency of the dominant component of the RESP series within the HF band and expressed as breaths per minute (bpm).

### Definition of CK^2^ and traditional K.^2^

A model-based spectral causality approach, namely CK^2^ [43, 44], was applied to assess the strength of the association in a specific time direction in the LF and HF bands. CK^2^ was computed because it is a directional technique, namely it can separate the dependence occurring on the feedforward pathway from that on the feedback one and it is spectral, thus allowing the evaluation of the influences between the two series in an assigned frequency band. CK^2^ was computed in the HF band from RESP to HP, namely ${\text{CK}}_{\text{RESP}\to \text{HP}}^{2}(\text{HF})$, and vice versa, namely ${\text{CK}}_{\text{HP}\to \text{RESP}}^{2}(\text{HF})$, to characterize the cardiorespiratory interactions, and in the LF band from SAP to HP, namely ${\text{CK}}_{\text{SAP}\to \text{HP}}^{2}(\text{LF})$, and vice versa, namely ${\text{CK}}_{\text{HP}\to \text{SAP}}^{2}(\text{LF})$, to describe the cardiovascular interactions. HP-RESP dynamic interactions were computed in the HF band because they comprise the time scales of RESP [40, 41], while HP-SAP dynamic interactions were computed in the typical band of the baroreflex functioning [37, 38]. CK^2^ computes the strength of the causal relationship from the cause to the effect by assessing the degree of squared association in closed loop and, then, by forcing to zero the effect of the pathway from the effect to the cause [43]. Markers increase above 0 with the strength of the association from the cause to the effect. The procedure followed to compute CK^2^ was fully described in [44]. Briefly, the technique is based on a description of the dynamic relations between the two series as a bivariate AR (BAR) model. Identification procedure was applied to linearly detrended HP, SAP, and RESP series. BAR model coefficients and the variances of the prediction errors were estimated by solving the traditional least squares problem via the Cholesky decomposition method [34]. The BAR model order was estimated via the Akaike figure of merit for multivariate processes within the range between 5 and 14 [46]. The latency from RESP to HP was set to 0 beats and from HP to RESP to 1 beat [24]. The latency from SAP to HP was set to 0 beats and from HP to SAP to 1 beat [37]. CK^2^ markers can be computed directly from the estimated coefficients of the BAR model and prediction errors [43, 44]. The CK^2^ markers were computed by averaging the CK^2^ functions in the LF, or HF, band.

Traditional noncausal K^2^ was computed as the ratio of the squared modulus of the power cross-spectral density between the two series to the product of their power spectral densities [39] computed using the same identification procedure utilized to obtain CK^2^ [43, 44]. The K^2^ function was averaged in LF, or HF, band as well to derive ${\text{K}}_{\text{RESP},\text{HP}}^{2}(\text{HF})$ and ${\text{K}}_{\text{HP},\text{SAP}}^{2}(\text{LF})$. Given the symmetry under reversal of the role of the two series, ${\text{K}}_{\text{RESP},\text{HP}}^{2}\left(\text{HF}\right)={\text{K}}_{\text{HP},\text{RESP}}^{2}\left(\text{HF}\right)$ and ${\text{K}}_{\text{HP},\text{SAP}}^{2}\left(\text{LF}\right)={\text{K}}_{\text{SAP},\text{HP}}^{2}(\text{LF})$ hold.

A normalization procedure was applied before computing CK^2^ and K^2^ such as way that variability series could exhibit zero mean and unit variance [43, 44]. This normalization was carried out by subtracting to each value the mean and by dividing the result by the standard deviation.

### Surrogate data approach

The significance of the K^2^ and CK^2^ markers was tested against a situation of full uncoupling between the two series generated via a surrogate data approach. Surrogate couples were built by a procedure preserving the distribution and power spectral density of the original series [49], but destroying their cross-correlation [50]. Cross-correlation was destroyed by substituting the Fourier phases of the original realization with random numbers drawn from a uniform distribution between 0 and 2π. The iteratively-refined, amplitude-adjusted, Fourier transform-based procedure was exploited to generate a surrogate series from the original one [49]. The procedure preserved exactly the original distribution of the series, while the power spectral density was the best approximation of the original one given 100 iterations. The use of two different random seeds to generate the sequences of random phases ensured that the surrogate pairs were uncoupled [50]. We generated 100 surrogate pairs from any original couple. We selected original sequences of 256 values to speed up the construction of the surrogate sets via fast Fourier transform procedure. K^2^ and CK^2^ markers were computed over each set of surrogates and the 95th percentile was extracted. If the marker computed over the original series was above the 95th percentile of the marker distribution built over surrogates, the null hypothesis of uncoupling was rejected and the alternative hypothesis, namely the series were significantly associated in the considered time direction, was accepted. The percentage of subjects with a significant K^2^, or CK^2^, was monitored as well and indicated as %K^2^ or %CK^2^. We computed ${\text{\%K}}_{\text{RESP},\text{HP}}^{2}(\text{HF})$ and ${\text{\%K}}_{\text{HP},\text{SAP}}^{2}(\text{LF})$ from traditional K^2^, and $\%{\text{CK}}_{\text{RESP}\to \text{HP}}^{2}(\text{HF})$, $\%{\text{CK}}_{\text{HP}\to \text{RESP}}^{2}(\text{HF})$, $\%{\text{CK}}_{\text{SAP}\to \text{HP}}^{2}(\text{LF})$, and ${\text{\%CK}}_{\text{HP}\to \text{SAP}}^{2}(\text{LF})$ from CK^2^.

### Statistical analysis

One-way analysis of variance (Tukey test for multiple comparisons) was applied to interval demographic and clinical variables to check the difference across noCPAP, CPAP and IMV groups. If normality test (Shapiro–Wilk test) and equal variance test (Brown–Forsythe test) were not passed, Kruskal–Wallis one-way analysis of variance on ranks was applied. The χ2 test was applied to categorical demographic and clinical variables. The level of significance of each test was lowered according to the number of comparisons (*i.e.*, 3) to account for the multiple comparison issue.

Two-way repeated measures analysis of variance versus a reference (Holm–Sidak test for multiple comparisons, one factor repetition) was utilized to assess the significance of the differences of K^2^, or CK^2^, markers between groups (*i.e.*, noCPAP, CPAP, and IMV) within the same experimental condition (*i.e.*, REST or STAND) and between experimental conditions within the same group. The references were markers computed in the noCPAP group and at REST. If normality test (Shapiro–Wilk test) and equal variance test (Brown–Forsythe test) for the application of the Holm–Sidak test were not passed, the Mann–Whitey rank sum test, or the Wilcoxon signed rank test, was applied when appropriate. The level of significance of each test was lowered according to the number of comparisons (*i.e.*, 7) to account for the multiple comparison issue.

The traditional χ2 test, or McNemar’s test when appropriate, was applied to the proportion of subjects featuring the rejection of the null hypothesis of uncoupling to assess the effect of STAND within the same group, and the impact of COVID-19 severity within the same experimental condition. Even in this case comparisons were set as previously and the level of significance of each test was lowered correspondingly.

Statistical analysis was performed with a commercial statistical software (Sigmaplot v.14.0, Systat Software, San Jose, CA, USA). The level of statistical significance of all the tests was set to 0.05. A type-I error probability *p* smaller than the level of significance, eventually corrected according to the number of comparisons, was always taken as significant.

## Data Availability

The data presented in this study are available on request from the corresponding author. The data are not publicly available because they contain sensitive personal information.

## Abbreviations

COVID-19: Coronavirus disease 2019
SARS-CoV-2: Severe acute respiratory syndrome coronavirus 2
K^2^: Squared coherence
CK^2^: Causal K^2^
AR: Autoregressive
BAR: Bivariate autoregressive
ECG: Electrocardiogram
AP: Arterial pressure
HP: Heart period
SAP: Systolic AP
RESP: Respiratory signal
LF: Low frequency from 0.04 to 0.15 Hz
HF: High frequency from 0.15 to 0.4 Hz
ICU: Intensive care unit
CPAP: Group who underwent continuous positive airway pressure
noCPAP: Group who did not undergo either continuous positive airway pressure or invasive mechanical ventilation
IMV: Group who underwent invasive mechanical ventilation in ICU
ANS: Autonomic nervous system
REST: At rest in supine position
STAND: Active standing
